# Cell line authentication: a necessity for reproducible biomedical research

**DOI:** 10.15252/embj.2022111307

**Published:** 2022-06-27

**Authors:** Nicole Y Souren, Norbert E Fusenig, Stefanie Heck, Wilhelm G Dirks, Amanda Capes‐Davis, Franca Bianchini, Christoph Plass

**Affiliations:** ^1^ International Journal of Cancer Heidelberg Germany; ^2^ Leibniz‐Institute DSMZ − Deutsche Sammlung von Mikroorganismen und Zellkulturen Braunschweig Germany; ^3^ CellBank Australia, Children's Medical Research Institute The University of Sydney Westmead NSW Australia; ^4^ Division of Cancer Epigenomics German Cancer Research Center (DKFZ) Heidelberg Germany

**Keywords:** Cell line cross‐contamination, misidentified cell lines, good scientific practice, *International Journal of Cancer*, research integrity, Cancer, Science Policy & Publishing

## Abstract

Immortalized or continuous cell lines are invaluable tools in basic and preclinical research. However, the widespread use of misidentified cell lines is a serious threat to scientific reproducibility. Based on the experiences of mandatory cell line authentication at the *International Journal of Cancer* (*IJC*), we provide an overview of the issues pertinent to misidentified cell lines and discuss available solutions. We also summarize the lessons learned, revealing that at least 5% of the human cell lines used in manuscripts considered for peer review are misidentified. About 4% of the considered manuscripts are rejected for severe cell line problems, and most are subsequently published in other journals. In order to diminish such malpractice and its consequences for the scientific record, we postulate that strict multi‐layered quality control is essential. Besides journals and publishers, we encourage scientists, research institutions, and funders to take action on the matter and revise their respective policies. Hence, we provide concrete recommendations on introducing regular authentication schemes and staff training, and discuss future steps for enhancing good cell culture practices.

## Graphical abstract

This commentary discusses multi‐layered quality control of immortalized cell lines as an essential prerequisite for reliability of the scientific record and good cell culture practices.

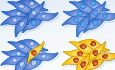

## Introduction

In 1951, the first immortalized or continuous human cell line, HeLa, was established by George Gey and colleagues using cervical adenocarcinoma cells originating from a patient named Henrietta Lacks (Sodeke & Powell, 2019). The HeLa cells were distributed around the world and numerous continuous cell lines have since been established from human cancers and normal human tissues. As continuous cell lines have the ability to proliferate infinitely, they are extensively used as model systems to study the molecular origins of human disease, and to discover and develop effective drugs. Due to large‐scale profiling initiatives, like the Cancer Cell Line Encyclopedia, multi‐omics data (including genomics, epigenomics, transcriptomics, proteomics, and metabolomics data) of more than 1,000 cancer cell lines are already publicly available (Ghandi *et al*, 2019; Nusinow *et al*, 2020), providing scientists with a wealth of information for direct analyses or for selecting the best models for their research.

**Figure 1:**
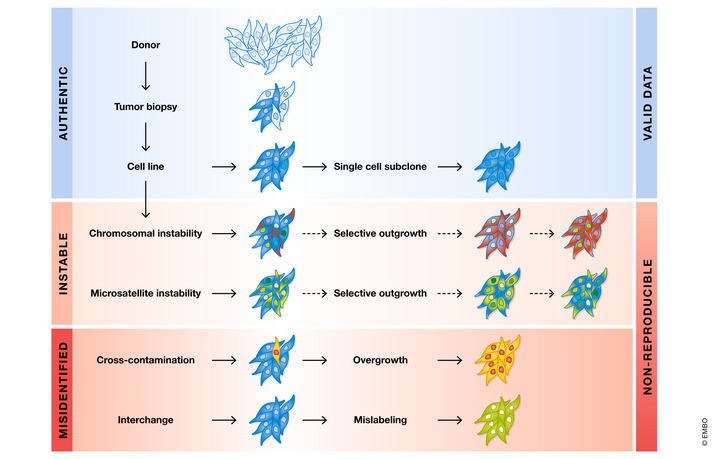
Scenarios of data generation using cell lines as tumor models The vast majority of scientific data are produced using authenticated cell lines, which generally reproduce well (blue area). Genetically unstable cell lines with highly heterogeneous populations may be subject to drift due to changing culture conditions (dashed arrows), making reproducible results difficult (light red area). Misidentified cell line model systems are most critical (red area) because reproducible data are virtually impossible to achieve. In the case of known misidentified cell lines, *i*.*e*., listed in the register of misidentified cell lines with no known authentic stock, generated data can be reproduced, but are meaningless.

Continuous cell lines will continue to be important tools in biomedical research, but to guarantee meaningful and reproducible results, good cell culture practice is a prerequisite (Geraghty *et al*, 2014). Increasing reliance on published datasets means that the source of the data—the cell lines themselves—must be reliable. Unfortunately, too often poor laboratory practice, like inadvertent cross‐contamination or mislabeling, results in misidentified cell lines that no longer correspond to their original donor (Fig 1) (Capes‐Davis *et al*, 2010). Already in 1968, Stanley Gartler recognized the problem of misidentified cell lines, and published that 19 supposedly independent human cell lines were actually HeLa cells, including the presumed laryngeal carcinoma HEp‐2 and the embryonic intestinal cell line Intestine 407 (Gartler, 1968). Since then many studies unmasking misidentified cell lines have been published (Nelson‐Rees & Flandermeyer, 1977; MacLeod *et al*, 1999) (partly reviewed by (Hughes *et al*, 2007)), and in 2010, a register of known misidentified cell lines was set up (Capes‐Davis *et al*, 2010). In 2012, the International Cell Line Authentication Committee (ICLAC) was established, which is a voluntary group of scientists who maintain the register of misidentified cell lines, and aim to increase the awareness of the problem and promote authentication testing (Masters, 2012). Currently, the ICLAC register counts 576 misidentified cell lines (last release June 2021), including 531 misidentified cell lines with no known authentic stock and 45 partially contaminated cell lines where some stocks are misidentified but authentic material is known to exist.

Although various methods are suitable for authentication testing (Castro *et al*, 2013; Yu *et al*, 2015; Zhang *et al*, 2020), short tandem repeat (STR) profiling is the international reference standard for cell line authentication (Masters *et al*, 2001). This inexpensive technique is implemented by cell banks worldwide, has a great discriminatory efficiency, and is highly reproducible due to the availability of standardized kits (Capes‐Davis *et al*, 2013). In 2012, the American Type Culture Collection Standards Development Organization (ATCC SDO) Workgroup ASN‐0002 developed a standard for authentication of human cell lines by STR profiling, published by the American National Standards Institute. After revision in 2021, the standard includes detailed protocols for DNA extraction, STR profiling, data analysis, quality control of the data, and provides support in interpreting STR results in case of allelic drop‐outs by loss of heterozygosity or multi‐allelic gains due to microsatellite instability (ANSI/ATCC ASN‐0002‐2021, 2021).

The next major step forward in the fight against misidentified cell lines was web‐based authentication using STR search engines with linked STR reference databases (Dirks *et al*, 2010), and most importantly the inception of the Cellosaurus database by Amos Bairoch and his team in 2012. The Cellosaurus is a knowledge resource that aims to document all cell lines used in biomedical research (Bairoch, 2018), and provides already a wealth of information on more than 102,000 human cell lines (current release March 2022). Moreover, the Cellosaurus warns the user when a cell line is known to be problematic (*e*.*g*., misidentified, partly contaminated, misclassified), and assigns a unique Research Resource Identifier (RRID) to all cell lines as it participates in the Resource Identification Initiative (Bandrowski *et al*, 2015). In 2019, the Cellosaurus was expanded by the Cellosaurus STR similarity search tool (CLASTR), which enables researchers to compare their obtained STR profiles with those available in the Cellosaurus database (Robin *et al*, 2020). The current Cellosaurus release contains STR profiles of more than 8,000 distinct human cell lines, and is by far the largest cell line STR profile database. Taken together, all prerequisites, including a wealth of knowledge, robust authentication methods, comprehensive databases, and tools, that enable researchers to detect misidentified or cross‐contaminated cell lines are in place and easily accessible.

## The impact of misidentified cell lines

Despite all of these developments, whether knowingly or unknowingly, researchers continue to publish data based on misidentified cell lines. More than 50 years after Stanley Gartler showed that HEp‐2 is a derivative of the cervical adenocarcinoma HeLa cell line (Gartler, 1968), we at the *International Journal of Cancer* (*IJC*) still receive manuscripts in which HEp‐2 is used as a supposed laryngeal carcinoma cell line. Philippe Gorphe identified 1,036 articles published between 1954 and January 2018 specifically referring to the wrong laryngeal origin of HEp‐2 cells. He also showed that the number of manuscripts using the HEp‐2 cell line as a laryngeal carcinoma has increased over the past three decades (Gorphe, 2019). In 2017, Horbach and Halffman identified 32,755 articles reporting on research with known misidentified cell lines, which were again cited by approximately half a million other papers. The 32,755 articles were identified by searching for cell line names in the title, abstract, and keywords (Horbach & Halffman, 2017). In our experience, cell line names are rarely mentioned in these searchable fields, leading us to believe that these 32,755 articles are only the tip of the iceberg.

To quantify the damage of misidentified cell lines, Korch and Capes‐Davis tried to estimate the financial consequences of usage of the two HeLa contaminated cell lines HEp‐2 and Intestine 407 (Korch & Capes‐Davis, 2021). They concluded that roughly $990 million were spent to publish 9,894 manuscripts in which these two cell lines were used. As the ICLAC register currently counts 531 misidentified cell lines with no known authentic stock, it is likely that billions of research dollars have already been spent on studies using misidentified cell lines. Considering on top the amount of money that has been spent on subsequent studies based on those misidentified cell line papers, the total damage becomes tremendous (Korch & Capes‐Davis, 2021).

Data obtained with misidentified cell lines in preclinical cancer research might be used as a basis for developing or improving novel therapies for a specific organ and/or tumor type not represented by the misidentified cell line used. Thus, in addition to the obvious economic damage, data based on misidentified cell lines could misguide and delay therapy development, resulting in missed opportunities to improve human health.

## The prevalence of misidentified cell lines

The studies mentioned above only focus on the impact of known misidentified cell lines. However, cell line cross‐contaminations can occur in any cell culture laboratory and often go undetected. A retrospective analysis of the German Collection of Microorganisms and Cell Culture (DSMZ), including 848 leukemia–lymphoma cell lines received between 1990 and 2014 from 290 laboratories in 23 countries, showed a difference in the prevalence of cell line cross‐contamination depending on the cell line source. Among cell lines obtained from primary sources (*i*.*e*., from the investigators who established the cell line or from certified cell line banks), cell line cross‐contamination decreased from 15 to 6%. However, among cell lines obtained from secondary sources (*e*.*g*., the neighboring laboratory), the cross‐contamination prevalence remained high at 14–18% over the 25‐year timespan, indicating that one in six of the secondarily sourced cell lines, shared between laboratories, is misidentified (Drexler *et al*, 2017).

In 2015, the China Center for Type Culture Collection (CCTCC) raised alarm bells when they reported a misidentified cell line rate of 25% after profiling 380 cell lines obtained from 113 independent sources (Ye *et al*, 2015). Furthermore, among the cell line models originally established in China, they observed a misidentified cell line rate of 85.5% (59 of 69), comprising 30 distinct cell lines that were almost exclusively contaminated by HeLa or a suspected hybrid with HeLa and an unknown cell line (Ye *et al*, 2015). Two additional reports published in 2017 confirmed the high prevalence of misidentified cell lines in China (20.5 and 46%, respectively) (Bian *et al*, 2017; Huang *et al*, 2017). In a recent study of the CCTCC, the authors claim that the situation in China has started to improve, with a misidentified cell line rate of 24.1% in 2019 (Gu *et al*, 2022). However, the observed improvement is mostly due to the increasing number of cell line samples received from company labs, while the misidentified cell line rate among samples received from Chinese hospitals remained high (∼45%) (Gu *et al*, 2022).

## Lessons learned from mandatory cell line authentication at the *International Journal of Cancer*

One possibility to enforce cell line authentication in research laboratories is to ask for appropriate documentation from authors prior to publication of their data, as implemented in the editorial process at the *IJC*. Back in 2007, Roland Nardone called for action in a widely distributed “white paper”, in which he proposed that cell line authentication should be a condition for receiving research grants and for publishing cell line‐based research findings (Nardone, 2007). As a reaction, the editors of the *IJC* tried to establish a consortium with leading journals and funding agencies requiring mandatory cell line authentication as a prerequisite for publication and funding. Unfortunately, the approached journals and funding agencies did not join the initiative because they considered that the responsibility to avoid publishing data based on misidentified cell lines lies with the authors and the reviewers (Fusenig *et al*, 2017). Nevertheless, in 2010, the *IJC* started requesting from authors proper authentication of all human cell lines used in a manuscript at submission (Lichter *et al*, 2010). Before a manuscript is sent out for peer review, the cell line authentication documents, which should not be older than 3 years, are thoroughly verified by a qualified editorial team member to ensure that all human cell lines used in the manuscript are authentic (see Box 1). The *IJC* was among the first journals that implemented a cell line authentication policy. Since then many journals such as *Nature*, *Cell Press*, the *American Association for Cancer Research*, and *EMBO Press journals* have followed, but their policies are less strict. Due to the stringent cell line authentication procedure implemented at the *IJC*, we realize that the misidentified cell line problem is still very prominent. In order to get a precise picture, we have recorded detailed cell line‐related information from manuscripts considered for peer review that included original human cell line data and were submitted to the *IJC* between July 2018 and June 2021.

Box 1 **Summary of the cell line authentication policy implemented at the *IJC*.^a^**

• Authors must provide cell line authentication documents that are not older than 3 years of all continuous human cell lines used in their manuscript.^b^
• STR profiling is the preferred method for cell line authentication. Authors can perform STR profiling in their own laboratory or use the service provided by a laboratory or cell bank with certified quality control. Either way, the cell line authentication documents should include high‐quality electropherograms.
• Of continuous human cell lines obtained within the last 3 years from a commercial source, which guarantees cell line authenticity through in‐house quality control measures (*e*.*g*., ATCC, DSMZ), the corresponding purchase orders or invoices are accepted for authentication.
• The *IJC* also requests authentication of human cell lines for which no reference STR profile is available. The obtained STR profile should be compared to a public database (*e*.*g*., Cellosaurus), and should show that the cell line is unique and not cross‐contaminated or misidentified.
• The *IJC* also accepts single nucleotide polymorphism (SNP) based cell line authentication reports from service providers with certified quality control, but only for cell lines of which an SNP‐based reference profile is publicly available.
• Authors of studies describing the establishment of new human cell lines are strongly encouraged to include the summarized STR results in the manuscript for future reference.
• Since May 2019, the following information must be included in the Materials and Methods section:
  ○ All cell lines used must be listed using the official cell line name and its Research Resource Identifier (RRID) as available in the ExPASy Cellosaurus database (*e*.*g*., HeLa (RRID:CVCL_0030)).
  ○ The source/supplier of all cell lines used must be provided.
  ○ A statement confirming that all human cell lines have been authenticated using STR (or SNP) profiling within the last 3 years.
  ○ A statement confirming that all experiments were performed with mycoplasma‐free cells.

^a^Adapted from https://onlinelibrary.wiley.com/page/journal/10970215/homepage/ForAuthors.html#AUCEL.

^b^Until May 2019 cell line authentication documents not older than 4 years were accepted.

### At least 5% of cell lines in manuscripts considered for peer review are misidentified

**Figure 2:**
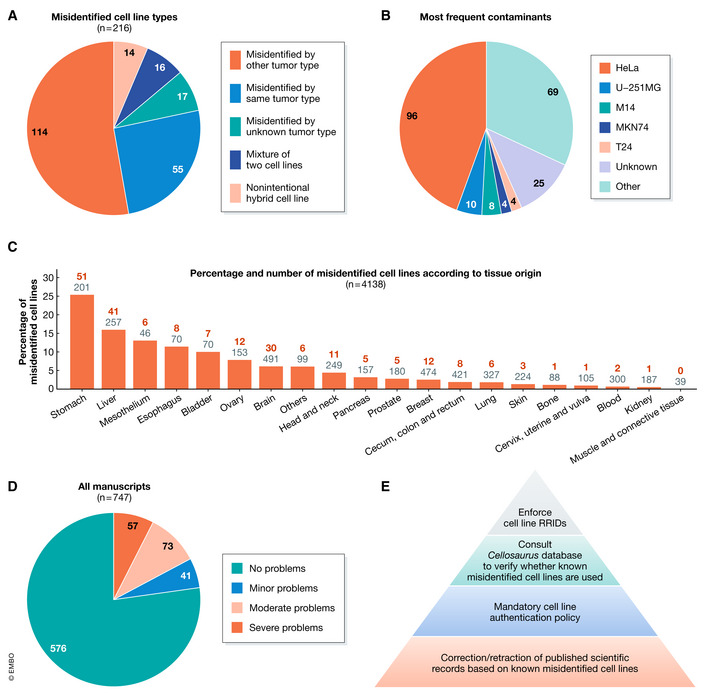
Characteristics of the cell line problems observed in 747 manuscripts considered for peer review that included original human cell line data ADifferent types and numbers of the 216 identified misidentified cell lines.BThe most frequently observed contaminants. A contaminant was reported as unknown if the contaminant was of non‐human origin, had a unique STR profile, was not reported, or not identified (*i*.*e*., SNP profiling).CPercentages and numbers of misidentified cell lines grouped according to the tissue origin (number of misidentified cell lines/number of cell lines in total).DOverview of the cell line‐related problems observed in the 747 manuscripts that were considered for peer review and included original human cell line data. Categories: Minor problems, *e*.*g*., minor textual adaptations; Moderate problems, *e*.*g*., one cell line had to be removed from the manuscript because it was misidentified; and Severe problems, *e*.*g*., at least two cell lines had to be removed from the manuscript because they were misidentified. See also Box 2.ERecommendations of editorial measures to avoid publishing studies based on misidentified cell lines and to correct the contaminated published records.

In this 3‐year period, 747 manuscripts containing original data on 4,138 human cell lines were considered for peer review. The majority of these submissions (∼65%) were not accompanied by valid cell line authentication documents and had to be unsubmitted to ask for correct documents, indicating that regular authentication of cell lines is not common practice. Eventually, valid authentication documents were obtained from 3,091 (74.7%) cell lines. By verifying the available cell line authentication documentation and cross‐checking the Cellosaurus database, 216 (5.2%) misidentified cell lines were identified, including 186 misidentified cell lines that were completely overgrown and replaced by a contaminator cell line, 16 mixtures of two cell lines, and 14 reported nonintentional hybrid cell lines (Fig 2A). The most frequent contaminants were HeLa (44.4%), U‐251MG (4.6%), M14 (3.7%), MKN74 (1.9%), and T24 (1.9%) (Fig 2B). Of the 216 misidentified cell lines, 126 (58.3%) are (now) known misidentified cell lines with no known authentic stock as recorded in the Cellosaurus database.

The highest percentage of misidentified cell lines was observed among gastric cancer (25.4%), followed by liver cancer (16%) cell lines (Fig 2C). Hence, our results indicate that the literature on these cancer types probably suffers a lot from misidentified cell line data. As listed in Table 1, the HeLa contaminated gastric or liver cancer cell lines BGC‐823, L‐02, SMMC‐7721, SGC‐7901, MGC‐803, and BEL‐7402 were among the most frequent misidentified cell lines. These cell lines were originally established by Chinese researchers and are to the best of our knowledge exclusively used in manuscripts from Chinese groups. While it was already reported in 2015 that these cell lines are misidentified by HeLa (Ye *et al*, 2015), in light of China now being the world's largest producer of scientific articles (Tollefson, 2018), this bad scientific practice has widespread consequences.

**Table 1:** Misidentified cell lines^a^ most frequently used in studies submitted to the *IJC* and considered for peer review.

Cell line name	Frequency^b^	Misidentified by	Original tumor/tissue type	True tumor type
BGC‐823	15	HeLa	Gastric carcinoma	Cervical carcinoma
L‐02	15	HeLa	Normal fetal liver	Cervical carcinoma
SMMC‐7721	15	HeLa	Hepatocellular carcinoma	Cervical carcinoma
SGC‐7901	14	HeLa	Gastric carcinoma	Cervical carcinoma
MGC‐803	12	Hybrid with HeLa	Gastric carcinoma	Cervical carcinoma^c^
MDA‐MB‐435	6	M14	Breast carcinoma	Melanoma
U‐373MG ATCC	6	U‐251MG	Astrocytoma	Astrocytoma
BEL‐7402	5	HeLa	Hepatocellular carcinoma	Cervical carcinoma

^a^ All cell lines are (now) registered in Cellosaurus as misidentified cell lines.

^b^ Only misidentified cell lines with a frequency ≥ 5 are listed in this table.

^c^ Hybrid with HeLa and a cell of unknown origin.

### About 4% of the manuscripts are rejected for severe cell line problems

Based on the available information, no cell line‐related problems were observed in 77.1% of the considered manuscripts (Fig 2D). However, minor, moderate, and severe cell line‐related problems were observed in 5.5, 9.8, and 7.6% of the manuscripts, respectively (see Fig 2D and Box 2). In total, 35 (4.7%) manuscripts were rejected for severe cell line‐related problems, including 6 manuscripts in which the obtained cell line authentication documentation was very clearly falsified (*e*.*g*., manipulated electropherograms or fake invoices).

Box 2 **Definitions of the different cell line‐related problems observed in the manuscripts. Related to Fig 2D.**

The cell line‐related problems observed in the manuscripts that were considered for peer review and included original human cell line data were divided into the following categories:

• *No cell line problems*: Based on the available cell line authentication documentation and by cross‐checking the Cellosaurus database, no cell line issues were observed.
• *Minor cell line problems*: These manuscripts only needed minor textual clarifications. For instance, the authors used a cell line that is known to be misidentified by another cell line from the same tumor type, as recorded in the Cellosaurus database, and the authors were asked to add this information to their manuscript (*e*.*g*., WiDr is a derivative of another colon adenocarcinoma cell line HT‐29). Or the authors were asked to clarify that two of the used cell lines originate from the same donor (*e*.*g*., the SW480 and SW620 cell lines are derived from the same patient).
• *Moderate cell line problems*: One cell line had to be removed from the manuscript because it was misidentified by a cell line from a different tumor type, the authentication documents revealed that the sample contained a mixture of two cell lines, or the cell line is recorded in Cellosaurus as a reported nonintentional hybrid cell line. Moderate cell line issues also include manuscripts containing misidentified cell lines that did not need to be removed because they are known to be misidentified by a cell line from the same tumor type as recorded in Cellosaurus, but the misidentified cell line had a large impact on the significance of the manuscript (*e*.*g*., a manuscript is entirely based on the three glioma cell lines U‐87MG ATCC, U‐251MG, and U‐373MG ATCC, but the U‐373MG ATCC is known to be a U‐251MG derivative, which was not mentioned in the original submission). Moderate cell line issues also include manuscripts in which a cell line needed to be renamed because the authentication documents showed that the cell line was misidentified by another cell line from the same tumor type, but the cell line was not among the known misidentified cell lines recorded in Cellosaurus.
• *Severe cell line problems*: At least two cell lines had to be removed from the manuscript because they were misidentified by a cell line from a different tumor type, the samples contained a mixture of two cell lines, and/or reported nonintentional hybrid cell lines were used. In case the misidentified cell line(s) affected a large amount of the presented data and/or the cell line authentication documentation was clearly falsified (*e*.*g*., authors uploaded electropherograms with intentionally falsely labeled alleles or provided obviously fake invoices [*i*.*e*., fabricated invoices listing known misidentified cell lines that are not distributed by ATCC as being purchased from ATCC]), then it was decided to reject the manuscript for severe cell line issues.

### Majority of manuscripts with severe cell line problems are published in other journals

In order to explore whether manuscripts rejected at the *IJC* with severe cell line‐related problems are subsequently published elsewhere, we followed up 63 manuscripts. These included 39 manuscripts from the analysis above that had been considered for peer review but were unsubmitted or rejected for serious cell line‐related problems between July 2018 and December 2020. The remaining 24 manuscripts that we followed up from this time period were *not* considered for peer review, but it was noticed that a large amount of the data were based on (known) misidentified cell lines. In all cases, the authors were clearly informed about the cell line problems observed in their manuscript in the unsubmission or rejection letter. Afterwards, we observed that 50 (79.4%) of these manuscripts were published in other journals, of which 45 (71.4%) manuscripts still included the data of the misidentified cell lines. Of these 45, 32 (50.8%) manuscripts included data of known misidentified cell lines with no known authentic stock, which means that the existing cell line problems could not have been solved in the meanwhile. Remarkably, in 5 (7.9%) cases, the data of known misidentified cell lines with no known authentic stock was renamed into another cell line. Additionally, one such manuscript was even resubmitted to the *IJC*. As the intentional publishing of misidentified cell line data represents scientific misconduct, the policy of the *IJC* is that new submissions of authors rendered guilty in this regard will not be considered. Because of data protection rules, the *IJC* cannot share personal data of authors with other journals. However, in order to correct the records, other journals are invited to contact the *IJC* to obtain general instructions on how they can identify published manuscripts that are based on known misidentified cell lines.

## Recommendations and perspectives for journals and publishers

Our experiences show that the misidentified cell line problem is very persistent and that strict editorial quality control procedures are essential to prevent publishing data based on misidentified cell lines. In order to avoid the publication of studies including misidentified cell line data and to correct the contaminated published records, journals and publishers should play a more active role. Hence, specific recommendations and perspectives for journals and publishers are discussed below and summarized in Fig 2E.

### Enforce research resource identifiers and consult the Cellosaurus database

Many commonly used cell line names are not unique (Bairoch, 2018). Hence, to ensure reproducibility it is important that cell lines are listed together with their research resource identifier (RRID). To obtain RRIDs, authors need to consult the Cellosaurus database or the RRID portal, both of which display a warning when a cell line is known to be problematic. As it has been shown that incidences of problematic cell lines are lower in manuscripts that use RRIDs to identify cell lines (Babic *et al*, 2019), enforcing the use of RRIDs might lead to fewer submissions of manuscripts containing data from known misidentified cell lines.

Moreover, about 60% of the misidentified cell lines identified in our evaluation are (now) known misidentified cell lines and are recorded in the Cellosaurus database. Therefore, journals can easily prevent publishing data based on these problematic cell lines by verifying all cell lines used in a manuscript in the Cellosaurus database even before starting the peer review process.

### Mandatory cell line authentication

Around 40% of the misidentified cell lines identified in our evaluation are not listed as known misidentified cell lines in the Cellosaurus database. This portion of misidentified cell lines can thus only be uncovered by implementing a strict mandatory cell line authentication policy. Verification of the authentication data by a qualified person is crucial because misidentified cell lines are often missed by authors due to misinterpretation of the STR results. We realize that verification of all cell line authentication documents will not be feasible for every journal. Nevertheless, if journals implement mandatory cell line authentication in combination with occasional verification of the documents, regular authentication of cell lines will become common practice and misidentified cell lines will be identified and eliminated earlier.

### Correction of scientific records

Even a strict cell line policy cannot completely prevent publishing manuscripts based on misidentified cell lines, and we encountered several cases where a correction of already published manuscripts with cell line problems became necessary.

Exemplarily, we obtained a unique STR profile of the presumed lung cancer cell line SPC‐A1, of which no reference STR profile was publicly available and the obtained STR profile did not give a match in the available STR databases. Two years later, we reassessed the STR profile in the updated Cellosaurus CLASTR tool, which revealed that the used SPC‐A1 cell line was actually the hepatocellular carcinoma MHCC97‐H cell line (Wu *et al*, 2020).

Further, we and others (Ye *et al*, 2015) noticed that several cell banks and companies in China are distributing HeLa contaminated cell lines (*e*.*g*., BGC‐823, SGC‐7901, SMMC‐7721), sometimes even accompanied by misleading cell line authentication reports. At the *IJC*, we accept purchase orders or invoices of authors who purchased their cell lines not longer than 3 years ago from commercial sources guaranteeing cell line authenticity through in‐house quality control measures, assuming that these sources would not distribute misidentified cell lines. Although our procedure has been adapted and invoices of such doubtful sources are now only accepted together with valid certificates of authentication, in the past manuscripts using these misidentified cell lines have been published and we are now in the process of correcting these records. However, publishing errata is a time‐consuming process, and there are hundreds of thousands of manuscripts published in other journals, based on known misidentified cell lines with no known authentic stock, that need to be corrected. Therefore, we support the idea of Horbach & Halffman (2017) who suggested a cross‐reference system between cell line databases, like the Cellosaurus, and scientific journal publications. It is time that publishers act and facilitate such an initiative.

### Paper mills

Nowadays, many biomedical journals, including the *IJC*, receive imitated manuscripts that are products of so‐called paper mills (Byrne & Christopher, 2020; Heck *et al*, 2021). We noticed that a strict mandatory cell line authentication policy helps in preventing publishing such paper mill manuscripts. Unfortunately, we also observed that paper mills are creative and fabricate or reuse (parts of) cell line authentication reports or certificates of analyses. It is therefore important to implement strict requirements and so we only accept high‐quality cell line authentication reports with original electropherograms. In order to prevent paper mills from gaining easy access to high‐quality cell line authentication data, we strongly advise against publishing cell line authentication data (*e*.*g*., electropherograms) as Supplementary Material.

### Mouse cell line authentication

The *IJC* also receives studies that use cell lines of nonhuman origin, among which mouse cell lines are the most common. Although the species of origin and inter‐species cross‐contamination can easily be detected using a multiplex PCR‐based assay (Yu *et al*, 2015), the development of methods to detect intra‐species cross‐contamination of mouse cell lines is more challenging. Due to intensive inbreeding, genetic heterogeneity is low within mouse strains, which complicates the identification of individual mouse cell lines (Almeida *et al*, 2014). Nevertheless, progress is being made, as in 2019, the Mouse Cell Line Authentication Consortium validated a multiplex PCR assay containing 18 mouse STR markers on 50 mouse cell lines (Almeida *et al*, 2019). Subsequently, the Cellosaurus extended the CLASTR tool to enable a similarity search using mouse STR profiles, and the current Cellosaurus release contains 77 mouse STR profiles. Unfortunately, a commercial kit for mouse cell line authentication is not (yet) available and only a limited number of companies are offering mouse cell line authentication at the moment. Therefore, authors submitting to the *IJC* are currently only highly encouraged to authenticate their mouse cell lines. Such cell lines can also be checked by testing for unique genomic features, such as specific mutations. The introduction of mandatory authentication of mouse cell lines is of high priority but should take place with the availability of appropriate techniques and service offerings.

## Recommendations for scientists and research institutions

Although journals and publishers play a crucial role in intercepting the publication of misidentified cell line data, scientists and research institutions have an equally important role to play in preventing the generation of such data by avoiding the use of misidentified cell lines. Our experiences indicate that regular authentication of cell lines is not common practice in everyday life and a radical change in the preparations of scientific projects is needed. Hence, we propose several recommendations for scientists and research institutions that would ensure cell line authentication becomes standard practice in research laboratories.

### Regular authentication of old and new cell lines

Cell lines in long‐term use should be authenticated at the beginning and the end of a project, especially after selection procedures (*e*.*g*., drug resistance, stable transfection), and whenever a phenotypic change—particularly in growth behavior—is observed. In general, scientists are advised to acquire continuous cell lines directly from certified cell banks that guarantee cell line authenticity through internal STR analyses (*e*.*g*., ATCC, DSMZ). If a cell line is obtained from a secondary source (*e*.*g*., fellow researcher), the cell line should be quarantined antibiotic‐free until its identity has been confirmed by STR profiling, and the overall sterility and absence of mycoplasma have been verified.

The establishment of new cell line models is an important but difficult task, as cross‐contamination at the source is one of the most common causes for the development of misidentified cell lines (MacLeod *et al*, 1999). If good laboratory practice is not followed, there is a risk that primary cells will be contaminated with cells from a foreign cell culture that have already been immortalized. Therefore, once sustained growth of the culture is achieved, an STR profile should be generated and preferably be cross‐checked with the STR profile of the original tissue sample or compared to a database of cell line STR profiles (*e*.*g*., by using CLASTR). For future reference, summarized STR results should be included in the manuscript as an essential part of the initial description so that authentication data can be included in the Cellosaurus database and associated with the RRID.

### Training in good cell culture practice

In communication with authors, we generally notice that the awareness and knowledge about the problem of misidentified cell lines are low. Hence, research institutions should incorporate mandatory training on good cell culture practices into the education of young biomedical scientists. Basic training material is freely available on the ICLAC website (https://iclac.org/education/). For further and detailed instructions on good cell culture practice, we refer to the “Guidance document on good *in vitro* method practices (GIVIMP)” by the Organization for Economic Co‐operation and Development (OECD, 2018), and other best practice documents (Geraghty *et al*, 2014).

### Authentication policies by funding agencies and research institutions

In principle, guidelines for higher authentication rates of continuous cell lines can be applied at three levels: the funding agencies (whose funding provides the foundation for research projects), the research institutions themselves, and finally, the publication bodies. In this respect, funding agencies can play a very important role if they require proof of authentication for the planned use of tumor models to be submitted at the time of application, as already practiced by the German Wilhelm Sander Foundation, the US National Institutes of Health, or the British Cancer Research UK.

At the *IJC*, we notice that authentication of cell lines is a more common practice among researchers who are affiliated to research institutions with an own dedicated cell line authentication facility. This type of authentication infrastructure has long been established at large companies such as AMGEN, Merck, or Roche Holding, but is also increasingly provided at large research institutions such as the German DKFZ or MD Anderson Cancer Center in the USA. Internal STR profiling facilities allow easy access to rapid authentication testing and ensure the presence of in‐house expertise for the interpretation of challenging samples, such as hybrid cell lines or mixtures. Hence, research institutions should consider offering a cell line authentication service in their own core facility. In addition, biomedical research institutions should develop guidelines for cell line use, as implemented by the MD Anderson Cancer Center requiring all researchers to authenticate their cell lines once a year. For a draft, we refer the reader to Schweppe and Korch, who suggested a detailed cell line and tissue authentication policy for biomedical research institutions (Schweppe & Korch, n.d.). Moreover, manuscripts from authors affiliated with research institutions having an institutional cell line authentication policy will definitely benefit if the authors confirm in the Materials and Methods that they have followed the institutional policy.

## Conclusions

Twenty years ago, when John Masters and colleagues introduced STR profiling as the international reference method for human cell line authentication, they were very optimistic and stated: *“This analysis could become a prerequisite for publication*, *so that the problem of cell line cross‐contamination can be reduced to a minimum in the future”* (Masters *et al*, 2001). Unfortunately, we have to face the fact that we still have a bumpy road ahead of us, which varies in length from country to country. Despite the strict cell line rules implemented at the *IJC*, still at least 5% of the human cell lines used in manuscripts considered for peer review are misidentified and about 4% of the considered manuscripts are rejected for severe cell line problems. The fact that the majority of the manuscripts unsubmitted or rejected for severe cell line problems have since been published in other journals, illustrates the irresponsibility of some authors toward the misidentified cell line problem. Hence, we call on scientists, research institutions, journals, and publishers to take action by implementing a mandatory cell line authentication policy and by developing a strategy to correct the contaminated literature. A collaborative approach that includes proactive funding agencies, rapid access to low‐cost authentication services, and widely requested proof of authentication from journals should significantly reduce the chronic dilemma of using misidentified cell lines in the future.

## Author contributions

**Nicole Y Souren:** Conceptualization; data curation; formal analysis; investigation; visualization; writing – original draft; writing – review & editing. **Norbert E Fusenig:** Conceptualization; supervision; writing – review & editing. **Stefanie Heck:** Conceptualization; data curation; investigation; writing – review & editing. **Wilhelm G Dirks**: Expert advice; visualization; writing – review & editing. **Amanda Capes‐Davis**: Expert advice; writing – review & editing. **Franca Bianchini:** Supervision; project administration; writing – review & editing. **Christoph Plass:** Conceptualization; supervision; project administration; writing – review & editing.

## Disclosure and competing interests' statement

The authors declare that they have no conflict of interest.
